# Catheter-Related Right Atrial Endocarditis in a Dialysis Patient

**DOI:** 10.7759/cureus.52144

**Published:** 2024-01-11

**Authors:** Maria Inês Ribeiro, Francisco D'Orey, João Prosil Sampaio, João Grade Santos, Vera Pereira

**Affiliations:** 1 Intensive Care Department, Hospital Garcia de Orta, Almada, PRT; 2 Faculty of Medicine, Universidade Católica Portuguesa, Lisboa, PRT; 3 Intensive Care Department, Hospitall Garcia de Orta, Almada, PRT; 4 Cardiology Department, Hospital Garcia de Orta, Almada, PRT

**Keywords:** transoesophageal echo, echocardiogram (echo), chronic kidney disease, hemodialysis access, infective endocarditis, right atrial mass

## Abstract

Hemodialysis catheters are frequently used for vascular access in end-stage chronic kidney disease patients lacking mature arteriovenous fistula. The incidence of infective endocarditis in hemodialysis patients is higher than in the general population and is associated with severe and potentially life-threatening complications. A high index of suspicion is imperative for early diagnosis and timely intervention to enhance the prognosis of this high-mortality condition. Imaging studies, like transthoracic and transesophageal echocardiography, are crucial for prompt diagnosis.

We present a case of a 36-year-old woman undergoing hemodialysis, whose prolonged use of a permanent catheter led to the development of infective endocarditis complicated with septic pulmonary embolism. Our case report presents an example of right atrial endocarditis with a poor outcome due to delayed diagnosis.

## Introduction

Patients with chronic kidney disease who do not yet have a mature arteriovenous fistula often require the placement of long-term central venous access for hemodialysis [1]. Despite efforts to reduce the risks, the prolonged use of central venous catheters correlates with an elevated risk of potentially life-threatening complications, including thrombosis, intravascular catheter-related bloodstream infections, and infective endocarditis (IE) [2].

Left-sided endocarditis is significantly more frequent, but a long-term central venous catheter is a risk factor for right-sided infective endocarditis [3]. Septic pulmonary embolism is a common complication and is associated with poor prognosis [4]. Therefore, early identification and prompt intervention are crucial for improving outcomes. Imaging studies play a pivotal role in the diagnosis of intracardiac masses, offering valuable insights for timely medical or surgical treatment [5].

In this case, we aim to highlight the critical importance of recognizing potential life-threatening complications associated with extended hemodialysis catheter use. The presented patient’s prolonged catheter dependence led to the development of a large IE mass with a poor outcome.

## Case presentation

We present a 36-year-old woman with a past medical history of a congenital malformation syndrome resulting in a neurogenic bladder, hydrocephalus, and colonic dysfunction. Presently, she suffers from end-stage renal disease requiring hemodialysis. Due to previous episodes of arteriovenous fistula thrombosis hemodialysis was performed via a permanent dialysis catheter inserted through the right internal jugular vein six months earlier.

She was admitted to the hospital following an outpatient transthoracic echocardiography (TTE), which detected a right atrium (RA) mass. On admission, she complained of dyspnea and orthopnea and she was febrile, hypotensive, and tachycardic. Peripheral oxygen saturation was 90% on room air.

Laboratory tests revealed normocytic anemia of 6.2 g/dL, thrombocytopenia of 33x10^9 ^cells/L and a raised C-reactive protein of 26.98 mg/dL. A presumptive diagnosis of septic shock/sepsis was made, leading to the initiation of empiric intravenous vancomycin and ceftazidime after obtaining blood cultures (Table 1).

**Table 1:** Laboratory results at emergency room admission

		Reference range
Hemoglobin	6.2 g/dL	11.5-18.0
Hematocrit	19,3%	37-54%
Mean Cell Volume (MCV)	94.4 fL	76-96
Mean Corpuscular Hemoglobin (MCH)	30.4 pg	27-32
Platelets	33 x10^9^/L	130-400
WBC	10.5 x10^9^/L	4-11
Neutrophils	77.9%	40-74
Lymphocytes	13.6%	19-48%
Glucose	98 mg/dL	60-110
Urea	105 mg/dL	16-48
Creatinine	6.3 mg/dL	0,5-0,9
Sodium	136 mmol/L	135-145
Potassium	4.9 mmol/L	3.5-5.0
Aspartate aminotransferase (AST)	50 UI/L	<32
Alanine aminotransferase (ALT)	19 UI/L	<35
Lactate dehydrogenase (LDH)	959 UI/L	100-250
Troponin T High-sensibility	297 ng/L	<14
C-reactive protein (CRP)	26.98 mg/dL	<0.2

Electrocardiography showed sinus tachycardia. Repeat inpatient TTE demonstrated the large echogenic cardiac mass in the RA with prolapse through the tricuspid valve (Video 1). 

**Video 1:** Transthoracic echocardiogram four-chamber view The video demonstrates the vegetation within the right atrium prolapsing through the tricuspid valve.

**Figure 1:**
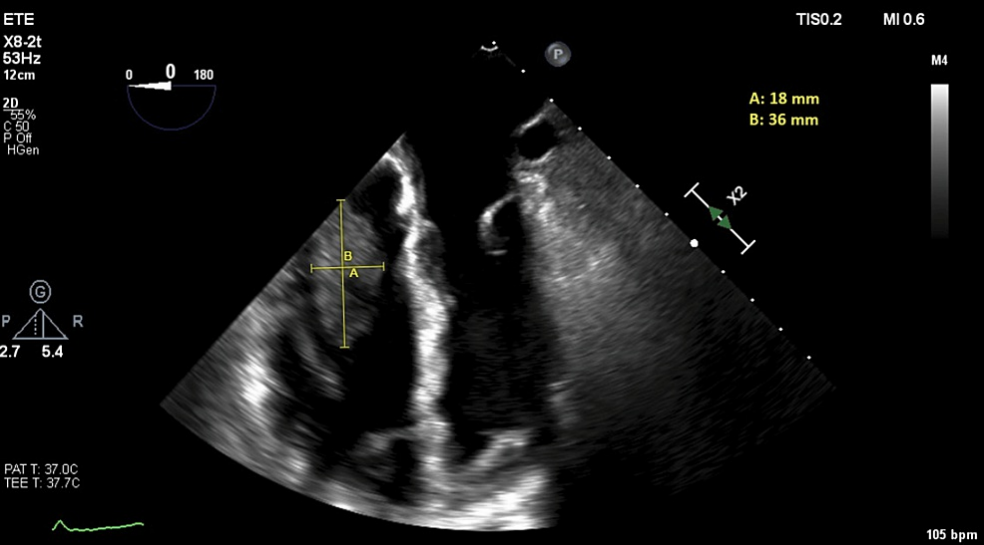
Transoesophageal echocardiogram The image shows the large irregular hypermobile right atrium mass (18 x 36 mm) adjacent to an indwelling catheter protruding through the tricuspid valve.

This finding was explored further by transesophageal echocardiogram (TEE) which confirmed the mass at the tip of the dialysis catheter in the superior vena cava outlet to the RA (Figure 1).

**Figure 2:**
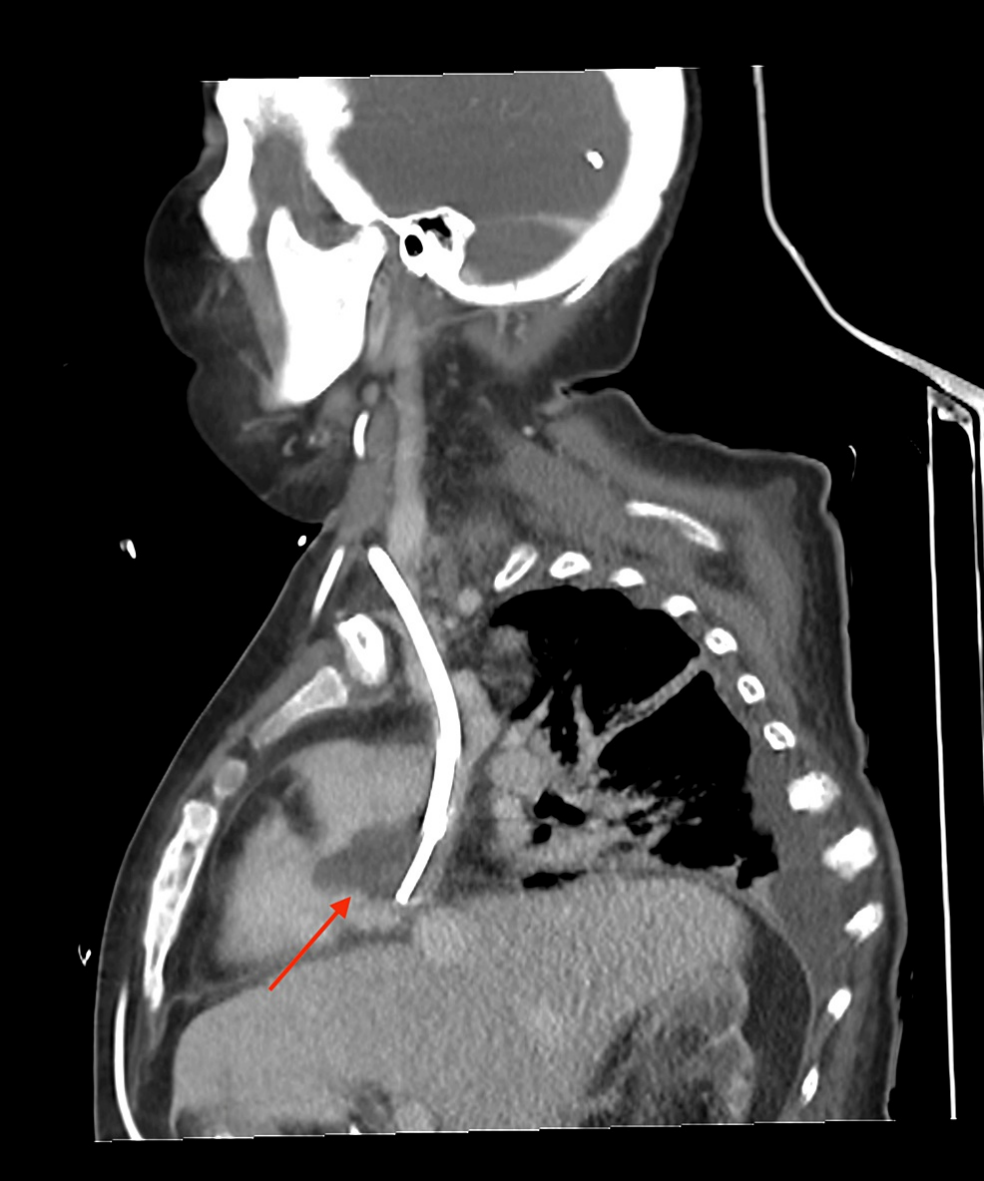
CT scan of the chest (sagittal view) Chest computer tomography shows the mass at the tip of the dialysis catheter into the right atrium (red arrow).

**Figure 3:**
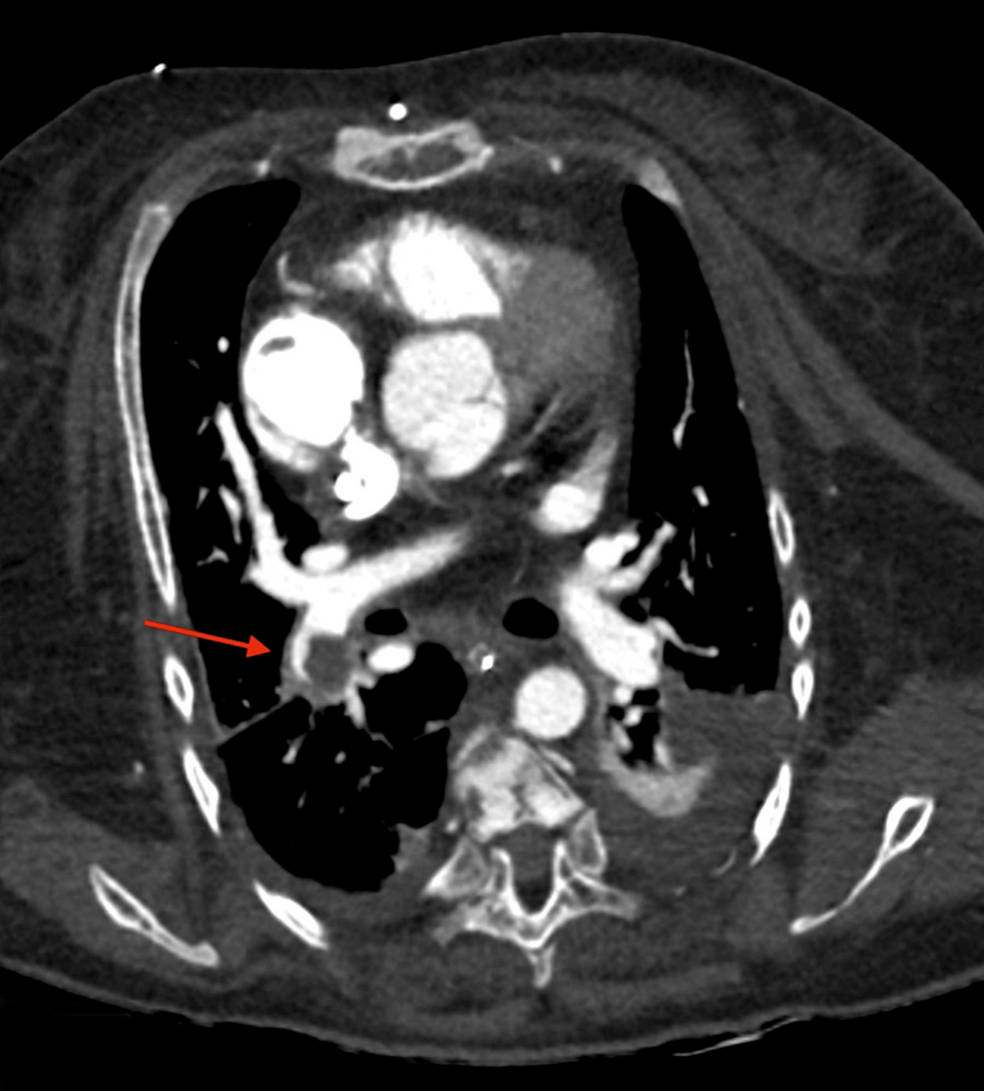
CT scan of the chest (axial view) Chest computer tomography shows the right segmental pulmonary embolism (red arrow).

Chest computerized tomography (CT) pulmonary angiogram confirmed the RA mass measuring 33 x 22 mm and showed a segmental pulmonary embolism (PE) (Figures 2, 3). 

CT head, abdomen, and pelvis revealed no further suspicious masses. Anticoagulation was not initiated due to severe thrombocytopenia and high hemorrhagic risk. A flucloxacillin-resistant *Staphylococcus capitis* was detected in the blood cultures and therefore she was continued on vancomycin alone. Based on all the clinical and radiological information and the Modified Duke criteria (fulfilling two major clinical criteria), she was diagnosed with infective endocarditis.

After four days, the patient deteriorated becoming more hypotensive and with altered consciousness. She was admitted to the intensive care unit (ICU) requiring mechanical ventilation, hemodynamic support, and monitoring. On the first 48 hours of ICU admission, she remained hemodynamically unstable, requiring fluid resuscitation and increased vasopressor dose. She showed a slow clinical improvement, allowing for the reduction of the dose of norepinephrine and ventilatory support.

After multidisciplinary discussion with cardiology, nephrology, intensive care medicine, and cardiothoracic surgery, catheter removal was deemed high risk due to potential embolization. Surgical removal of the mass was postponed to allow adequate antibiotic treatment and patient stabilization. On day 4 of ICU, she went into a state of refractory shock, prompting an increase in intravenous vasopressors. Piperacillin-tazobactam was added to the antibiotic therapy. Despite this treatment, there was no reduction in the vegetation size, and the patient rapidly developed multiple organ failure. Following another multidisciplinary review, surgical intervention was deemed not appropriate at that stage and the patient died on day 10 of hospital admission.

## Discussion

Although increasingly common, the prolonged use of long-term dialysis catheters is associated with severe infection and mechanical complications [6]. Atrial masses are uncommon but can pose significant management challenges with high mortality risk. The differential diagnosis of a right atrial mass is extensive and includes thrombus formation, bacterial vegetation, or cardiac tumor (most commonly atrial myxoma). TTE is the initial diagnostic imaging technique to characterize RA masses. Other complementary diagnostic imaging tests may also be useful to help characterize the mass, such as TEE, CT, and cardiac magnetic resonance [7]. Fluorodeoxyglucose Positron Emission Tomography (PET) is also useful to help differentiate between a cardiac mass and IE [8]. 

In this case, the presence of bacteriemia with septic shock, the size, form, and location of the mass starting at the tip of the catheter and progressing into the heart, and the fully met Modified Duke Criteria support the diagnosis of RA bacterial vegetation. Furthermore, one of the most common complications associated with right-sided IE is septic PE, which is likely to be the cause of this patient´s PE [4]. 

Anticoagulation was not provided to our patient due to severe thrombocytopenia, which posed an increased risk of hemorrhage. Additionally, while anticoagulation therapy is crucial for treating noninfective pulmonary embolism, it is generally avoided in cases of septic embolization due to the heightened risk of bleeding in the infected embolus area [9].

IE is significantly more common in hemodialysis patients than in the general population [10]. Left-sided endocarditis is more common and better documented than right-sided endocarditis, but patients using central venous catheters have an increased risk of developing right-sided IE. Common microorganisms causing IE in hemodialysis patients are *Staphylococcus aureus*, *Enterococcus* and *Streptococcus*. Gram-negative species and fungal agents are rarely found [11]. Aggressive management, including early cardiac surgery to remove the source of infection, is crucial. We believe that an earlier diagnosis of IE might have led to a better prognosis, preventing such a refractory shock. This, in turn, could have prompted early surgical intervention, potentially resulting in a more favorable outcome.

## Conclusions

Hemodialysis patients, particularly those with indwelling catheters, represent a high-risk population for IE, and its exclusion in the early workout of sepsis is paramount. Even with appropriate treatment, mortality remains high. Each situation is unique and requires multidisciplinary team discussion to determine the best treatment options. To improve the prognosis, a high index of suspicion is necessary for early diagnosis and timely intervention. Both TTE and TEE are the fundamental imaging techniques used to diagnose, manage, and follow cases of IE.
